# Daylight saving time and incidence ratio of acute myocardial infarction among Iranian people

**DOI:** 10.25122/jml-2017-0058

**Published:** 2019

**Authors:** Mani Mofidi, Nahid Kianmehr, Yaser Foroghi Qomi, Sonia N. Zaim, Peyman Hafezi Moghadam, Mahdi Rezai, Davood Farsi, Saeed Abbasi, Babak Mahshidfar

**Affiliations:** 1. Emergency Medicine Management Research Center, Iran University of Medical Sciences, Tehran, Iran; 2. Iran University of Medical Sciences, Department of Rheumatology, Hazrat Rasoul Akram Complex, Tehran, Iran; 3. Qom University of Medical Sciences, Qom, Iran

**Keywords:** Daylight Saving Time (DST), Acute Myocardial Infarction (AMI), circadian rhythm, sleep, circadian misalignment

## Abstract

Iran is among the countries which change official time, every year according to their constitutions. Studies have shown an increase of incidence ratio (IR) of acute myocardial infarction by these time transitions. Despite annual time changes in Iran, there is no published data to prove this among the Iranian. This retrospective study examined the IR of patients with AMI, who were admitted to the Emergency Department (ED) of 5 teaching hospitals during the week just after the time transitions (observed period), with two weeks before and after the time transitions (expected period), both in spring and fall. In total, 11051 patients were admitted during the ten weeks (observed and expected), in both spring and fall time transitions. The IR of AMI during both observed and expected period did not show any significant difference (p > 0.05); however, the incidence of AMI was increased during the first week after the transition in spring (p > 0.05). Although the results of the present study did not prove the relation between time transitions and incidence of AMI, a slight increase existed for IR of AMI during three days after spring shift. This increase in IR of AMI can be due to Nowrooz, the national holidays which lasts four days after turning clocks forward in Iran.

## Advances in Knowledge

To the best of our knowledge, this is the first study that evaluates the relationship between daylight saving time (DST) and acute myocardial infarction (AMI) in people in the Middle East.

The findings of this study showed a modest increase in AMI incidences during the first week following the spring transition.

## Application to Patient Care

The results of this study can indicate an increase in the rate of cardiac problems, suggesting some considerations to decrease these effects.

## Introduction

Time transition was officially used by Germany and Austria after the First World War in order to reduce energy consumption. These countries turned their clocks one hour forward in the period between April and October. This practice was gradually accepted by some other countries [1,2].

Circadian rhythm has been known for a long time in cardiac physiology. Some indicators underline the fact that heart functions such as blood pressure, heartbeats, heart muscle function and responses to the hormones have rhythmic patterns [3,4]. The sleep cycle is one of the most important examples of circadian rhythm. The adjustment to a 24-hour sleep cycle could be affected by environmental factors. Also, a normal response of the human body to sunset and darkness which results in lower temperature consists of decreasing body temperature and preparing it for sleep. Circadian rhythm can be disrupted by aging, sleep disorders and chronobiology [5,7].

From 1991 onward, Iran joined the countries practicing DST, the practice of setting clocks one hour forward from the standard time in spring, and back again in fall. Official time transition in Iran is practiced on spring and autumnal equinox. We need to mention that the first 4 days after the vernal equinox transition represent a national ceremony and holiday (Nowrooz) in Iran. This specific condition affects the relation between time transitions and incidences of AMI which seems reasonable to be considered [8]. To our knowledge, there is no published data about DST and incidence ratio (IR) of AMI amongst Iranian people.

## Methods

The subjects of this survey were the patients admitted to the emergency departments (EDs) of five teaching hospitals supervised by the Iran University of Medical Sciences (IUMS) and Tehran University of Medical Sciences (TUMS) during the 10 weeks of the study. The study was approved by the Research Ethics Committee of IR.IUMS.Rec.1395.9311307019.

All of the patients with the diagnosis of AMI were included in the study [9]. Patients who did not have documented electrocardiographic characteristics or elevated cardiac enzymes were excluded from the study. We compared the data obtained in seven days after the transition with the data obtained from the corresponding days of two weeks after and before these seven days.

The time specified for the study was the seven days after the time transitions to DST, March 20^th^ to 26^th^, the 2 weeks before the transition time, March 6^th^ to 19^th^, 2012 and the two weeks after March 26^th^. The same pattern was applied for autumnal equinox.

## Data Analysis

To assess the influence of DST on the incidences of AMI, the following parameters were defined:

Y=∑AMI/∑p

∑AMI: The sum of AMI in a day, ∑p: All patients admitted to the EDs in 24 hours; Y was calculated for each day of the observed and the expected period.

Observed group: The ratio of patients with the diagnosis of AMI of all the patients admitted to the EDs of the participating hospitals (AMI incidences), for each day of the first week after the transition.

Expected group: The ratio of all AMI incidences in each corresponding day of the 2 weeks after DST and two weeks before the time shift. For instance, on the first Wednesday after the transition, we divided the incidences that occurred on Wednesday by the average of the incidences that occurred on Wednesday two weeks earlier and the incidences that took place on Wednesday two weeks after the transition. Incidence ratio was determined as IR= Observed/Expected. We compared the AMI incidences in the observed period with the AMI incidences in the expected period.

The statistical analyses were performed using the Statistical Package for Social Sciences (SPSS) for Windows 18.0 (SPSS Inc., Chicago, IL, USA). Incidence ratios were compared using the OpenEpi module. In analytical statistics, nominal or ordinal variables were compared between groups using Fisher’s exact test, depending on the expected cell counts of the corresponding crosstabs. Statistical significance was set at p < 0.05.

## Results

A total number of 2392 patients were presented to the EDs of 5 university hospitals during the “observed week”. Thirty-one people out of the total were diagnosed with AMI. During the control (expected) period, all of the admitted patients to the EDs of the mentioned hospitals were 8659 and 111 of them were diagnosed with AMI. Baseline characteristics are shown in Table 1.

**Table 1:** Baseline characteristics of the enrolled patients in two groups

		Age (mean, 95%CI))	Gender (male)	History of ACS	Previous PCI or CABG	History of Cardiac Medication
**Observed**	MI	51.7 (47.8–56.2)	24 (77.4%)	10 (41.6%)	2 (8.3%)	12 (50%)
	Not MI	42.1 (33.9–50.8)	1578 (67.2%)	593 (25.1%)	95 (4.02%)	715 (30.28%)
**Expected**	MI	53.1 (49.2–56.4)	79 (71.71)	49 (44.14%)	10 (9%)	55 (49.54%)
	Not MI	39.4 (30.8–44.5)	5616 (65.7%)	2308 (27%)	261 (3.05%)	2111 (24.69%)

MI=Myocardial Infarction, CI=Confidence Interval, ACS=Acute Coronary Syndrome, PCI=Percutaneous Coronary Intervention, CABG=Coronary Artery Bypass Graft

## Spring Shift

There were no statistically significant differences in the number of AMI during these two periods in spring (p = 0.869).

The comparison between the incidence ratios of AMI during the observed week and the corresponding days of control (expected) period, showed a slight increase in the incidences for the observed period; but the differences were not significant statistically (p > 0.05).

## Autumn Shift

Tables 2 and 3 represent the incidence ratios of AMI after turning the clocks backward in spring and fall, respectively. According to the data, there was not any statistically significant relationship between time transition and AMI incidences in the autumn either (p = 0.861).

**Table 2:** Incidence Ratios (IRs) of acute myocardial infarction (AMI) during spring

			Spring				
**Day**	1^st^ day	2^nd^ day	3^rd^ day	4^th^ day	5^th^ day	6^th^ day	7^th^ day
**IR**	0.889	0.844	1.25	1.209	1.304	1.395	0.477
**95%CI**	0.883–0.894	0.838–0.849	1.241–1.258	1.201–1.217	1.292–1.315	1.381–1.408	0.465–0.465

**Table 3:** Incidence Ratios (IRs) of acute myocardial infarction (AMI) during fall

			Fall				
**Day**	1^st^ day	2^nd^ day	3^rd^ day	4^th^ day	5^th^ day	6^th^ day	7^th^ day
**IR**	0.823	1.385	1.266	1.119	1.04	0.71	1.652
**95%CI**	0.814–0.831	1.368–1.402	1.252–1.28	1.106–1.131	1.026–1.054	0.689–0.721	1.632–1.672

**Figure 1:**
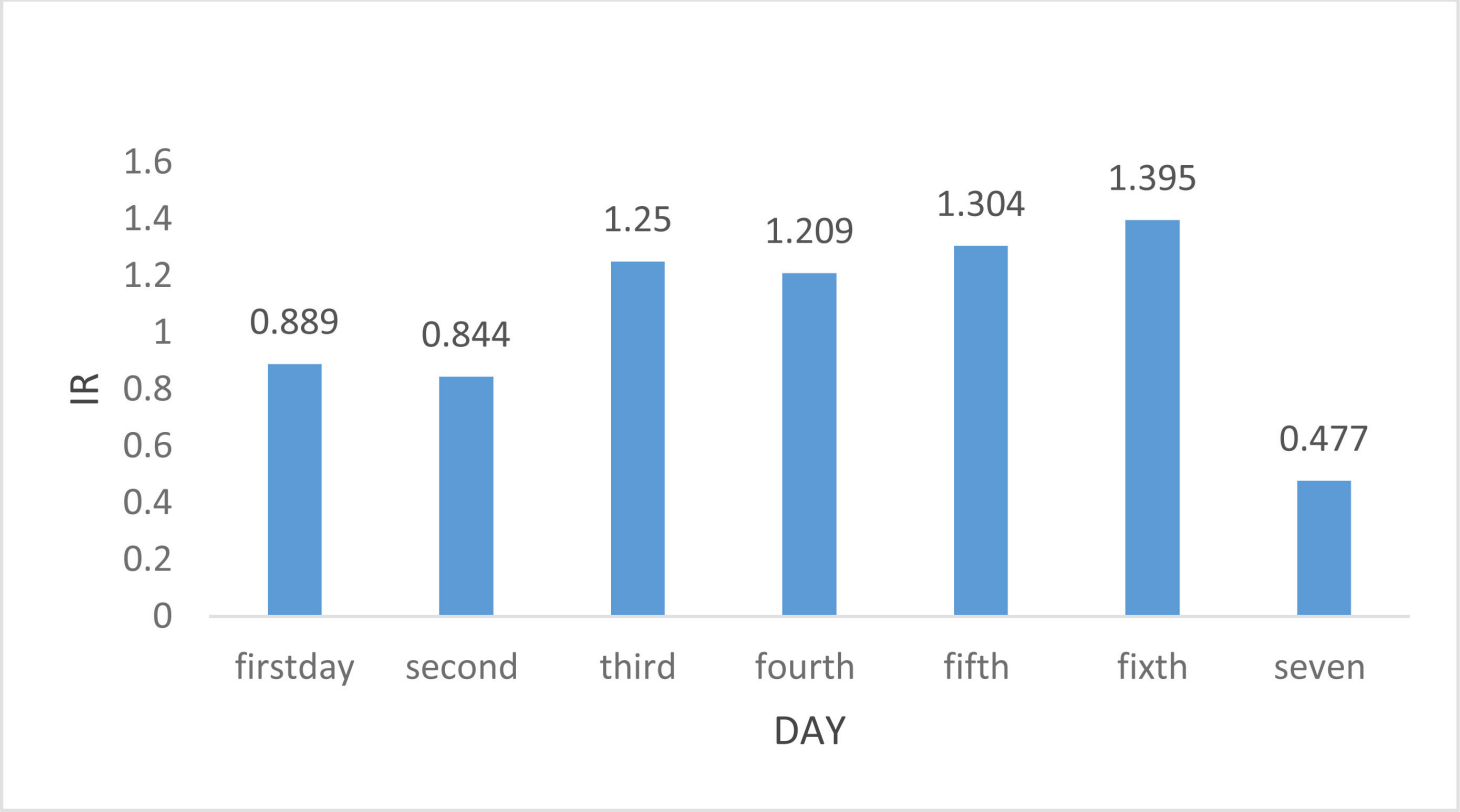
IRs of AMI in spring shift

**Figure 2:**
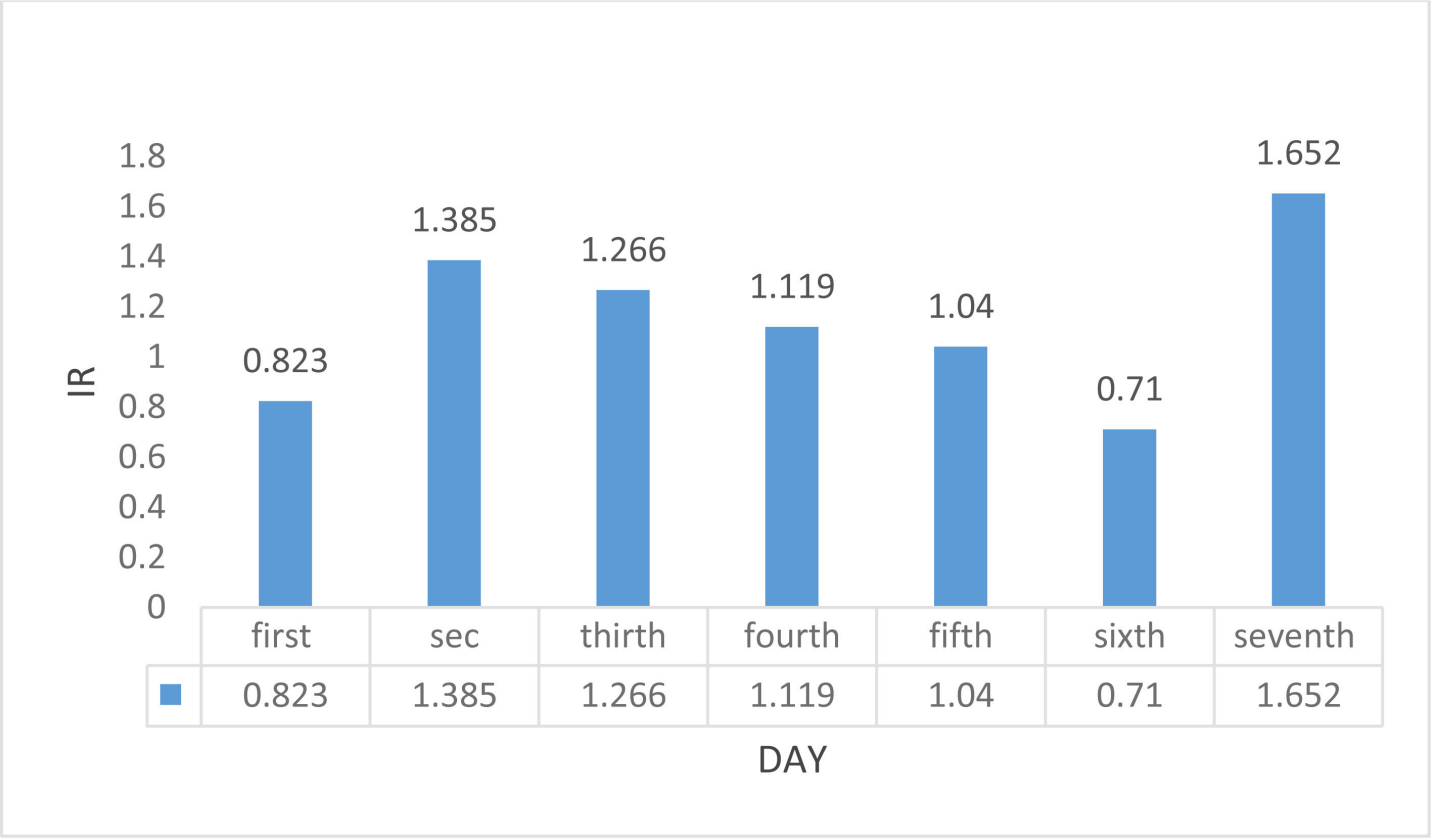
IRs of AMI in fall shift

Figure 1 and 2 present AMI incidence ratios during the first week after time transition in spring and fall, respectively. Figure 1 shows a slight elevation in IRs during the first week after setting the clocks forward. A slight decrease in IR was seen through the observed period in the fall.

## Discussion

We designed this study to assess the correlation between the official time transitions in Iran and the occurrence of AMI in a sample of population. The findings of this study showed that the ED visits were more frequent in spring compared to fall. In spring, the third day (544), and the seventh day (902) had the fewest and the most ED visits, respectively. The sixth day (109) and second day (1050) of fall had the least and the most ED visits, respectively.

The data shows a slight increase of IR of AMI in the observed period in spring shift; but, this rise did not reach a significant level.

Although there was not any statistically significant difference between IRs of AMI for the observed period of spring and fall shifts, the pattern which was observed in this study was the same as the previous studies. According to Janszky et al. [10] and Jiddou et al. [12], the IR of AMI increased in the first 3 weekdays after the spring transition [10,11]. The overall pattern for the observed week in spring was similar to the one from the mentioned studies; this applies for the fall shift as well. A slight decrease was found in the IRs of AMI during the fall transition. The findings of our study showed a different pattern for the seventh day in the observed weeks of both spring and fall. Despite the ascending pattern of IR of AMI during the first week after the spring shift, the ratio suddenly decreased (from 1.4 to 0.4) on the seventh day; the IR of AMI rose from 0.7 on the sixth day to 1.6 on the seventh day after the shift, during the observed period in fall. Jiddou reported similar findings [11]. In another study, the reported IRs of AMI were 1.04, 1.06, and 1.10 for the 3 weekdays, respectively [12]. This study showed smaller IRs of AMI (for instance, 0.8 vs. 1.06 for the second day). A recent study has shown an increased AMI risk after the DST in spring, especially in the first week after the shift but there was not any significant association in the first week after autumn [8]. These results are consistent with the findings of our study. It may suggest that circadian misalignment and disruption of sleep during the first week after the spring shift increases the risk of AMI.

Previous studies have found that the sleep-wake cycle needs to adjust to the transitions. If the body fails to get adjusted to this change, the sleep cycle and its efficacy might be disrupted. Some studies have explored several biological pathways by which acute sleep deprivation could stimulate coronary problems. Sleep deprivation causes sympathetic predominance, catecholamine release which results in increased heart rate and blood pressure [12]. Another study confirmed that even minor sleep deprivation might heighten pro-inflammatory cytokines levels such as interleukin-6, tumor necrosis factor and C-reactive protein [11]. One may speculate that such processes might result from the cortisol excess during the first week after the time transition, because of the sudden change in sleep habits; however, cortisol typically acts as an anti-inflammatory hormone and cortisol excess can indicate the presence of inflammation [13–16]. This condition puts the body at a higher risk for AMI.

Some studies have shown that the patients who used Angiotensin Converting Enzyme (ACE) inhibitors are more prone to be affected by time transitions. Besides, other studies found a relation between ACE gene function and cortisol secretion, a process that may be affected by cortisol excess [8, 16]. According to these facts, we can suggest that vulnerable people may benefit from gradual changes in the sleep cycle, instead of the acute changes. Further research is needed to explain this finding. For instance, the pattern of daily blood pressure variations during the observed period would reveal more information about the DST impact on the circadian rhythm (especially regarding the heart function or immune system).

Limitations of this study are similar to the previous studies on the same subject, such as the insufficient data for the sleep quality, the sleep chronotype (morning or evening type), the duration of sleep deprivation and emotional states. Also, latitude, the temperature, and the darkness may have an impact on the sleep-wake cycle. Additionally, confounding factors are the unavoidable problems of the retrospective studies; for instance, heart problems and drug history may affect the patients. Prior studies pointed out that their data did not include the verified AMI by electrocardiographic features or the rise of cardiac enzymes. In terms of strength, patients who did not have documented ECG or elevated cardiac enzymes were excluded.

## Conclusion

The findings of this study showed a modest increase in AMI incidences during the first week following the spring transition. One of the explanations for this situation is the misalignment of the circadian rhythm due to the sleep deprivation. Generalizing the findings of this study to all of the Iranian could be unreal since there may be other risk factors interfering with the results.

## Conflict of Interest

The authors confirm that there are no conflicts of interest.
